# Discovery, Biosynthesis, Total Synthesis, and Biological Activities of Solanapyrones: [4 + 2] Cycloaddition-Derived Polyketides of Fungal Origin

**DOI:** 10.1021/acs.jnatprod.4c00818

**Published:** 2024-11-15

**Authors:** Roberto G. S. Berlinck, Elizabeth Skellam

**Affiliations:** † Instituto de Quimica de São Carlos, Universidade de São Paulo, CP 780, São Carlos, São Paulo CEP 13560-970, Brazil; ‡ Department of Chemistry, BioDiscovery Institute, University of North Texas, Denton, Texas 76203, United States

## Abstract

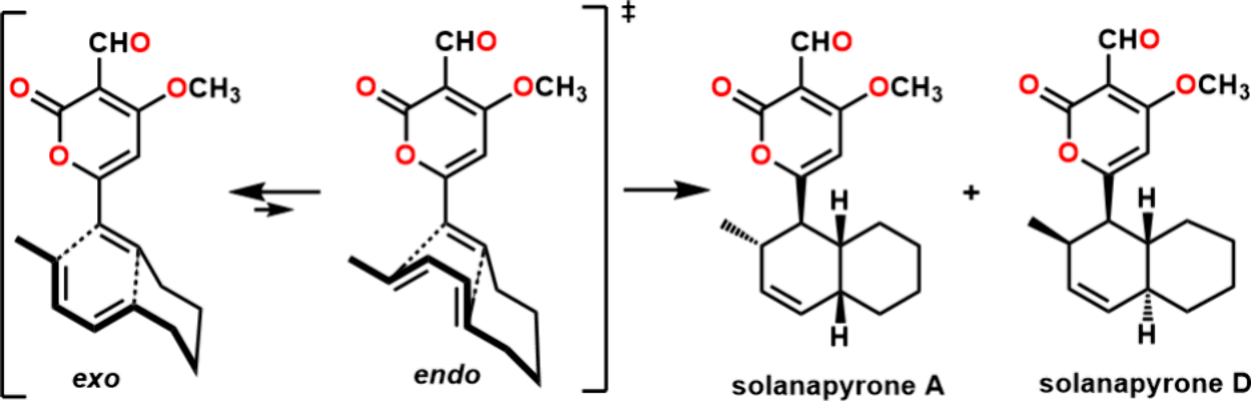

Solanapyrones are metabolites bearing a 3,4-dehydrodecalin moiety isolated from cultures of different fungi that are associated with plant diseases. Research on solanapyrones resulted in the first report of a Diels–Alderase enzyme implicated in natural product biosynthesis related to the formation of the 3,4-dehydrodecalin core. In addition, several total syntheses of solanapyrones have been reported, which are also connected with the formation of the characteristic cycloaddition-derived 3,4-dehydrodecalin moiety. This Review provides the first comprehensive overview on the chemistry, biosynthesis, and biological activities of solanapyrones under the theme of synthetic and biosynthetic research progress on cycloaddition-derived secondary metabolites.

Fungal secondary metabolites are of both historical and economic relevance; consequentially, they have multiple applications in the food industry and plant, human and animal health.^1^ Since antiquity, fungal-based medical preparations have been employed to treat various conditions, including tuberculosis and infections caused by pathogenic bacteria,^1^ but the characterization and production of fungal metabolites was accelerated only in the first half of the 20th century, such as, for example, the production of citric acid.^1^

The historical discovery of penicillin and its derivatives from cultures of *Penicillium* spp. was a landmark in medicine and for natural products chemistry, as it represented a new era in the search for new bioactive natural products, particularly antibiotics.^1,2^ Even though large screening programs were directed toward the investigation of actinomycetae antibiotics, fungi remained a reliable source of new bioactive compounds, ultimately leading to the discovery of compactin and its derivatives from cultures of *Penicillium brevicompactum* as the first natural inhibitors of 3-hydroxy-3-methylglutaryl coenzyme A (HMG-CoA) reductase.^3,4^ Also, several amines and alkaloids isolated from fungal cultures or from mushrooms display potent psychotropic mechanisms, such as psilocin from various mushroom species, muscimol from *Amanita* spp., lysergic acid derivatives from cultures of *Claviceps* and *Cordyceps*, and additional indole alkaloids from different fungi.^5^ These psychoactive compounds hold very promising therapeutic value and are under intense investigation and development for the treatment of neuropsychiatric disorders.^5^ Such major scientific breakthroughs keep fungi at the forefront of biological sources, providing unique biologically active secondary metabolites potentially useful for human health.

Solanapyrones are polyketide-derived fungal metabolites first reported in 1983 as phytotoxins produced in culture by the fungus *Alternaria solani*.^6^ Since the first report of solanapyrones A–C (**1**–**3**),^6^ a whole family of solanapyrones has been discovered from cultures of different fungal species. Solanapyrones are biosynthetically related to compactin and its derivatives because solanapyrones structurally hold a 3,4-dehydrodecalin moiety derived from an intramolecular [4 + 2] cycloaddition reaction. Because of the unique chemical scaffold and biological activities of solanapyrones, these compounds have been the targets of different total syntheses. In this short Review, we aim to discuss the isolation and identification of solanapyrones, as well as their biosynthesis, total synthesis, and biological activities, and to highlight the biosynthetic capability of distinct fungal strains to produce structurally related metabolites of biotechnological and biological interest.

## Isolation and Structure Analysis

**Figure 1:**
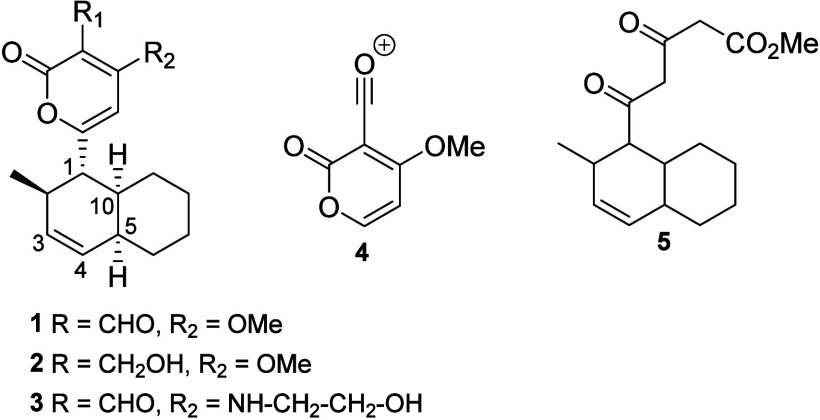
Structures of solanapyrones A–C (**1**–**3**) and distinctive fragments and degradation products identified during structure elucidation.

**Scheme 1:**
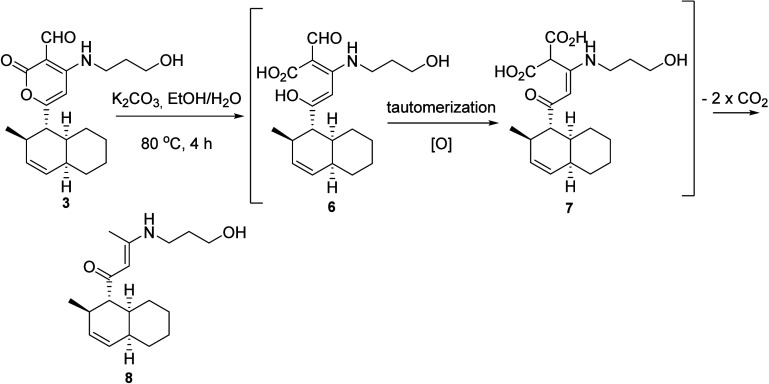
Degradation of the α-Pyrone Moiety of Solanapyrone C (**3**) via Decarboxylation

Solanapyrones A–C (**1**–**3**; Figure 1) were isolated from *A. solani* surface cultures.^6^ The presence of an α-pyrone ring was proposed based on UV, IR, and NMR analyses and was confirmed by electron impact mass spectrometry analysis, which indicated an ion at *m*/*z* 153 corresponding to the product ion with the formula C_7_H_5_O_4_^+^ (quite probably **4**; however, in the original communication by Ichihara et al.^6^ the ion value was indicated as *m*/*z* 158, probably as a typographical mistake). Further confirmation of the α-pyrone moiety in the structure of **1** was indicated by comparison with spectroscopic data reported in the literature, as well as by reacting **1** with KOH in MeOH/H_2_O to give the γ,β-diketo ester **5**. The remaining structural features were deduced based on the molecular formula and number of unsaturations, including a (*Z*)-double bond. Assignments for the dehydrodecalin moiety were established by ^1^H NMR selective decoupling experiments. The relative configuration at C-1, C-2, and C-10 was assigned based on the coupling constants observed for H-1 (9.8 and 11.7 Hz) with H-2 and H-10, respectively. The *cis*-bicyclic junction was deduced from the coupling constant observed between H-5 and H-10 (4.0 Hz). The structure of compound **2** was proposed by comparison with spectroscopic data recorded for **1** and also by acetylation (Ac_2_O, pyridine) to confirm the presence of the primary alcohol group, as well as by oxidation of the primary alcohol to the corresponding aldehyde (with pyridinium chlorochromate; PCC), to give compound **1**. The structure of **3** was proposed by comparison with data obtained for compounds **1** and **2**, as well as by (a) acetylation of **3** to give the corresponding acetyl ester at the hydroxy group of the ethanolamine residue and (b) hydrolysis of **3** with K_2_CO_3_ in EtOH/H_2_O at 80 °C, the product of which presented structure **8**, likely derived from decarboxylation of the intermediate **7** formed from **6** (Scheme 1).^6^

**Scheme 2:**
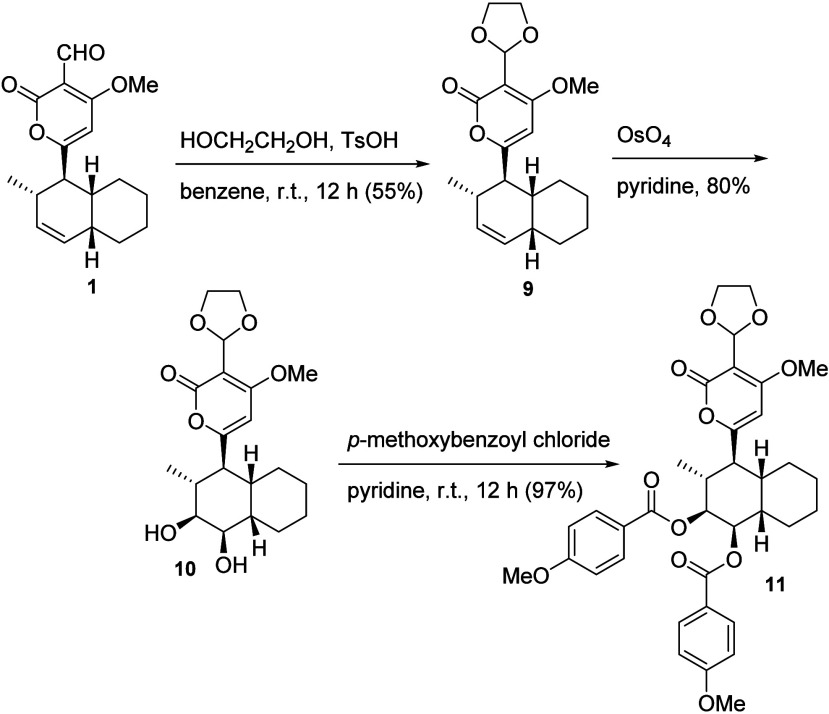
Determination of the Absolute Configuration of Solanapyrone A (**1**) by Chemical Derivatization to a Product Amenable to Electronic Circular Dichroism Analysis

The absolute configuration of solanapyrone A (**1**) was subsequently established by application of the circular dichroism exciton chirality method.^7^ Solanapyrone A (**1**) was converted into its cyclic ketal **9**, which was oxidized to its corresponding *cis*-diol **10** with the addition of aqueous OsO_4_ in pyridine. The diol **10** was then reacted with *p*-methoxybenzoyl chloride in pyridine. Circular dichroism of the reaction product displayed a negative first Cotton effect correlated to the absolute stereostructure **11**, corresponding to the absolute configuration (1*R*,2*S*,5*R*,10*R*)-**1** (Scheme 2),^7^ with the opposite configurational drawing for **1** (as well as for **2** and **3**) in its first isolation and identification report.^6^

The structures of solanapyrones A (**1**) and C (**3**) were further confirmed by 2D-NMR analyses and, in the case of solanapyrone C, by X-ray diffraction analysis, which indicated that the N–H hydrogen is intramolecularly bonded to the oxygen of the aldehyde carbonyl group. Since **1** and **3** share the same source and biosynthesis origin, the X-ray diffraction analysis also confirmed the absolute configuration of **1**.^8^

Solanapyrone D (**12**) was isolated as a diastereomer of solanapyrone A, probably from the same fungal strain (no experimental details were provided in the original report).^9^ The absolute configuration of **12** was established as performed for solanapyrone A (see above).^9^ The authors discussed the configurational assignment of **1** and **12** considering different outcomes of a possible intramolecular Diels–Alder cyclization as the key step for the formation of the dehydrodecalin bicyclic system (see below).^9^ The configuration assignment of solanapyrone D based on a biosynthetic hypothesis was subsequently confirmed by analysis of NMR data, in addition to chemical derivatization using the same approach used for solanapyrone A (**1**), as well as by using Mosher’s ester analysis.^10^ The Mosher’s derivatization occurred only at the equatorially oriented hydroxy group resulting from the dihydroxylation of **12**.^10^

In developing the synthesis of solanapyrones (see below), Oikawa and collaborators reported the isolation of the minor solanapyrone E, identified by comparison with the product of reduction of **12**.^11^ However, later on Jenkins and collaborators reported new solanapyrones, among which one was named as solanapyrone E.^12^ Because of the naming redundancy, herein we propose that the compound isolated by Oikawa et al.^11^ (**13**) should be named as solanapyrone E1, while the compound isolated by Jenkins et al.^12^ (**14**) should be named as solanapyrone E2. Solanapyrones E2–G (**14**–**16**) were isolated from cultures of an unidentified fungal strain coded CNC-159 obtained from the surface of the marine alga *Halimeda monile*.^12^ The structures of **14**–**16** were established by analysis of spectroscopic data, while the absolute configuration of solanapyrone F (**15**) was established by Mosher’s ester analysis.^12^

Solanapyrones A–C (**1**–**3**) were reisolated from cultures of the fungus *A. solani*, but the structures drawn in this communication were not correct in their depiction of the orientation for the methyl group at C-2 as well as the overall absolute configuration of **1**–**3**, which was previously established.^13^ Interestingly, the production yield of **1**–**3** was significantly higher in surface cultures than in liquid cultures of *A. solani*. The production yield of solanapyrone B (**2**) reached a maximum in 16 days of fungus growth, while the yield of **3** was maximal at day 17. After 18 days of the fungus growth, the production yield of solanapyrone A (**1**) was still increasing.^13^ Solanapyrone A (**1**) was later isolated from cultures of *Ascochyta rabiei*.^14^

While solanapyrones J–M (**17**–**20**) have been isolated from cultures of an unidentified fungicolous fungus, the authors cite a Ph.D. dissertation by O. Schlörke, (University of Göttingen, 2005) in which the isolation and structures of solanapyrones H and I were presented but not published.^15^ As reported in Dr. Schlörke’s Ph.D. dissertation, solanapyrone H was subsequently isolated and named as solanapyrone P (**26**, see below), while the structure of solanapyrone I (**21**) has not yet been published.^15^ The structures of **17**–**20** were established analysis of the spectroscopic data, including a detailed configurational analysis based upon NOE and ^1^H coupling constant information. Solanapyrone K was isolated as a mixture of co-occurring tautomers **18a** and **18b**, of which the minor one (**18a**) presents hydrogen bonding of the N−H hydrogen to the unsaturated ketone carbonyl group and the major tautomer (**18b**) has a hydrogen bond between the N−H hydrogen and the lactone carbonyl group. As for solanapyrone L (**19**), no specific configuration was suggested for the enamine group.^15^ Solanapyrones N (**22**) and O (**23a** and **23b**) were isolated from cultures of *Nigrospora* sp. YB-141, an endophytic fungus obtained from *Azadirachta indica*.^16^ Solanapyrone O was also isolated as a 5:1 mixture of tautomers (**23a** and **23b**, respectively), with data similar to those reported for the two tautomers of solanapyrone K,^15^ including the large coupling constant observed for the hydrogen at the enamine double bond (13 < *J* < 15 Hz), which was assigned to its coupling to the N–H hydrogen.^15,16^
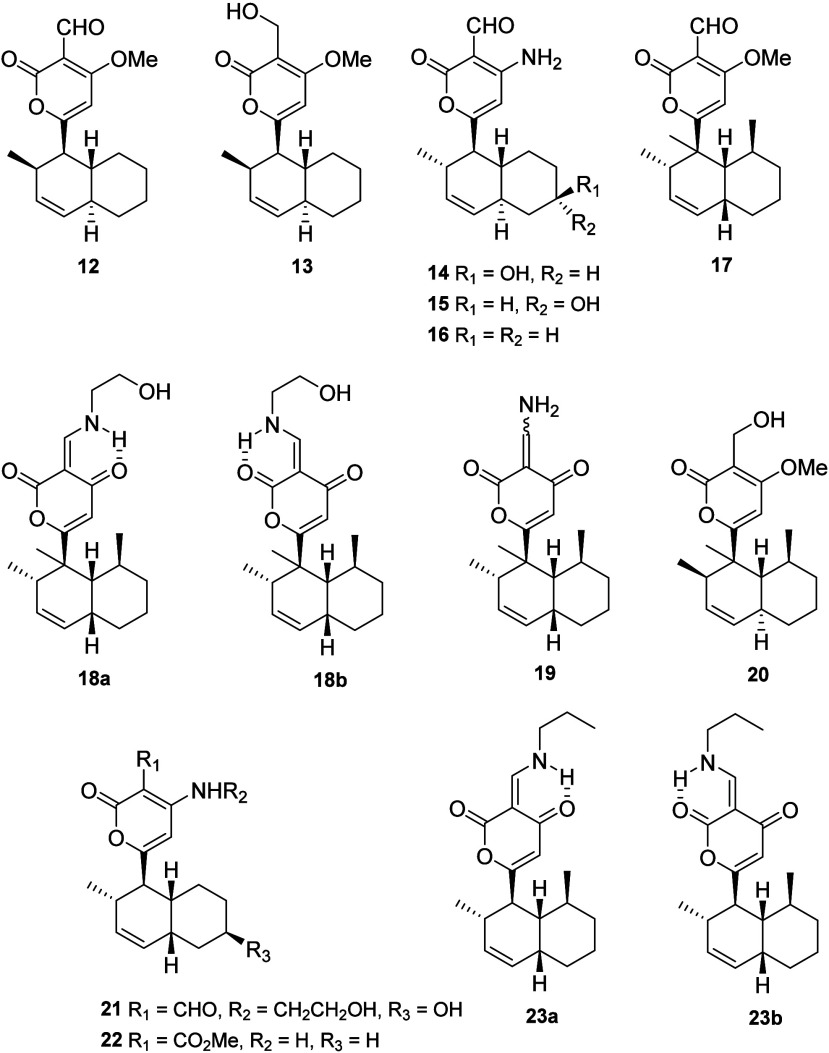


Nigrosporapyrones A–C (**24**–**26**) isolated from cultures of the marine-derived fungus strain *Nigrospora* PSU-F18 are closely related to solanapyrones.^17^ Solanapyrone A was also isolated and identified. Structure assignments for **24**–**26** were based on spectroscopic analysis. Interestingly, in order to explain the NOE correlations observed, the authors proposed a 3D conformation for nigrosporapyrones A (**24**) and C (**26**) in which the cyclohexene moiety of the dehydrodecalin system would adopt a boat-like conformation, while the cyclohexane moiety of the dehydrodecalin system would be in a chairlike conformation. However, during the preparation of this Review, we drew the proposed stereostructure for compound **24** and used the Chem3D 18.1.0.535 MM2 energy minimization protocol to test the hypothesis. The results of our analysis indicated that the cyclohexane moiety of **24** would adopt a boat like conformation with the hydroxy group oriented equatorially, while the cyclohexene moiety would adopt a semichair-like conformation, and the NOEs observed can also be explained by this result. The authors proposed that the chemical shift of H-5 (δ 2.59, m) in compound **26** would be higher than those observed for the same hydrogen in compounds **24** (δ 2.06, m) and **25** (δ 2.02, m) due to the steric compression of the axially oriented hydroxy group on H-5. A γ-gauche compression effect of the same axially oriented hydroxy group would also explain the chemical shift of C-5 (δ 30.4) in compound **26** when compared to the chemical shift of C-5 in compounds **24** (δ 44.1) and **25** (δ 44.2). Indeed, the authors’ claims are supported by similar results obtained for piperidine derivatives.^18^ Therefore, the use of Chem3D to support the NMR conformational analysis interpretation should be considered with caution.

Solanapyrones P–R (**27**–**29**) were isolated from cultures of *Alternaria tenuissima* SP-07 obtained from roots of *Salvia przewalskii*, which is used as an herbal medicine in China. The structures of **27**–**29** were established by analysis of spectroscopic data.^19^ Solanapyrones C (**3**), E2 (**14**), and G (**16**) and nigrosporapyrone B (**25**) were also reisolated from cultures of *Nigrospora* sp. YS7.^20^
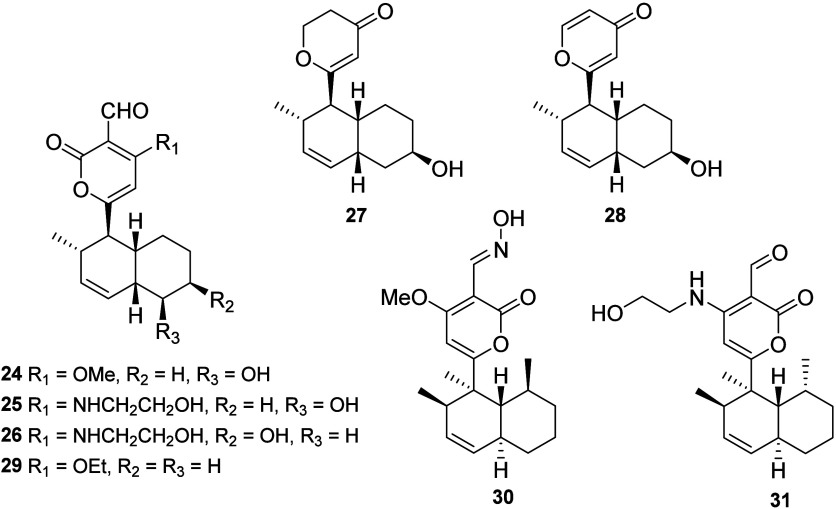


Two new solanapyrones S (**30**) and T (**31**) have been isolated from culture media of the fungus *Peroneutypa* sp., a strain obtained from the viscera of an unidentified sea cucumber (holothurian).^21^ Solanapyrone S features an oxime instead of a commonly observed aldehyde or, less frequently, a nitrogenated group, such as an amine in solanapyrones K (**18**) and O (**23**) or an enamine in solanapyrone L (**19**). The *trans*-fused dehydrodecalin bicyclic system is observed for both **30** and **31**, unlike the most frequent *cis*-fused dehydrodecalin for most solanapyrones. Structures of both **30** and **31** were established by analysis of the spectroscopic data, including ECD for the assignment of the absolute configuration.^21^

## Biosynthetic Investigations of Solanapyrones

**Scheme 3:**
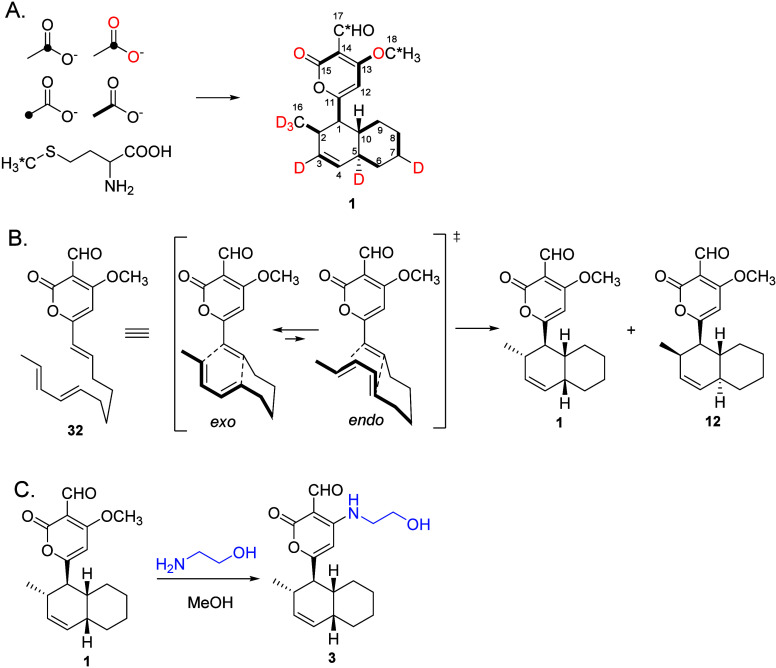
Summary of the Isotope-Labeled Incubation Studies Used to Investigate the Biosynthesis of Solanapyrones A (**1**) and D (**12**) and the Nonenzymatic Formation of **3**(10)

Initial investigations into the biosynthesis of solanapyrone A (**1**) by *A. solani* utilized isotopically labeled acetate and methionine to establish that **1** arises from an acetate-derived octaketide, whereas carbons C-17 and C-18 are introduced via methionine. These incorporation studies also established that the oxygen atoms at C-13 and C-15 originated from acetate (Scheme 3A).^22^ Further studies with isotopically labeled intermediates suggested that solanapyrone B (**2**) was a reduction product of solanapyrone A.^11^ In a separate investigation, the polyketide origin of solanapyrones A–C was confirmed in *A. rabiei* using isotopically labeled acetates as well; however, the results suggested that solanapyrone A (**1**) was an oxidation product of solanapyrone B (**2**).^23^ During the fungal production of **1** and **2**, related minor solanapyrones D (**12**) and E1 (**13**) were isolated and identified. Solanapyrones D (**12**) and E1 (**13**) are diastereomers of **1** and **2**, respectively, and are proposed as the *endo* products of the achiral triene intermediate prosolanapyrone III (**32**) (Scheme 3B). Incubation studies using isotopically labeled acetate suggested that **12** and **13** arise from the same biosynthetic pathway, which diverges at a later stage.^10^ The biosynthetic origin of solanapyrone C (**3**) was determined by treating **1** with ethanolamine *in vitro*, indicating a nonenzymatic nucleophilic addition mechanism (Scheme 3C).^10^

**Scheme 4:**
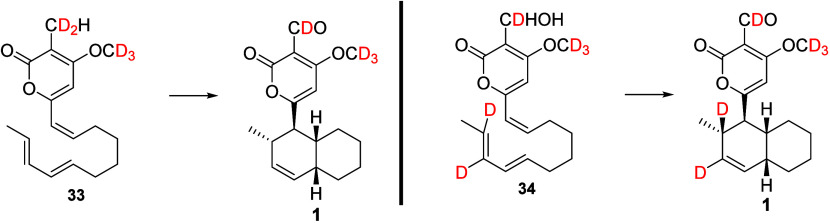
Advanced Isotope Labeling Studies to Understand the Biosynthesis of Solanapyrone A (**1**).^22^

The relative configuration of the decalin core in the solanapyrones and the position of the double bond at C3/C4 are suggestive of a biological intramolecular [4 + 2] cycloaddition reaction. This observation was further supported by the isotope labeling studies and the presence of minor amounts of the *endo* solanapyrones **12** and **13** identified from fungal cultures. To confirm that the decalin core formed via a [4 + 2] cycloaddition reaction, the deuterium-labeled achiral linear trienes **33** and **34** were synthesized and supplied separately to *A. solani*. Solanapyrone A (**1**) containing deuterium atoms was detected from both experiments; where **33** was supplied, **1** was determined to have incorporated deuterium atoms at C-17 and C-18, while where **34** was utilized deuterium atoms were detected at C-2, C-3, C-17, and C-18, suggesting intact incorporation of the precursors (Scheme 4).^22^ The observed high *exo* selectivity indicated that an enzyme was required, as chemical synthesis did not yield the same product profile.^24−27^

## Identification and Elucidation of the Solanapyrone Biosynthetic Gene Cluster

**Figure 2:**
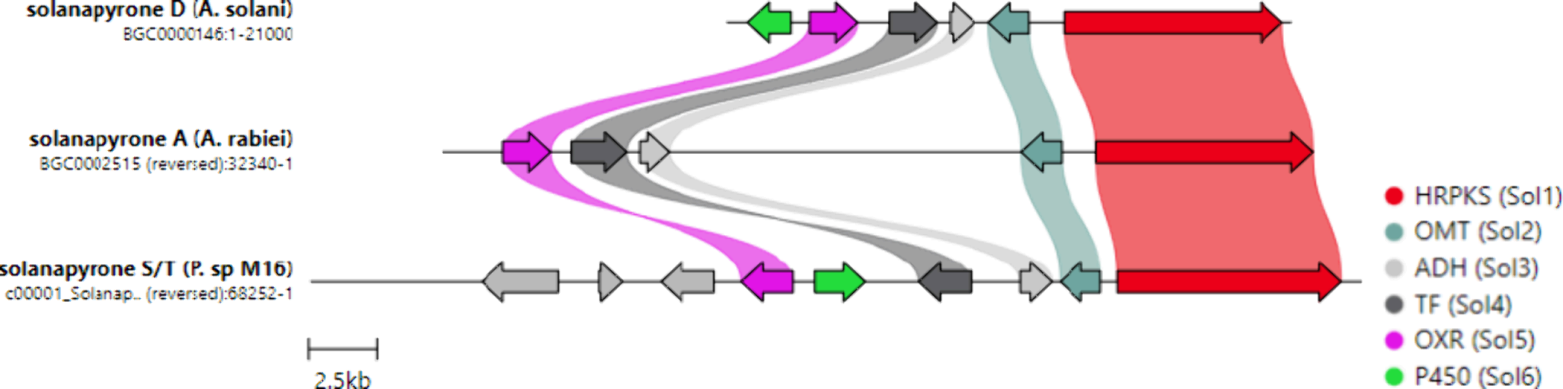
Comparison of biosynthetic gene clusters encoding solanapyrones. Data from Kim et al.^32^ and from Amorim et al.^33^

Although there had been extensive attempts to identify the enzyme required for the [4 + 2] cycloaddition reaction from cell-free extracts, proposed as solanapyrone synthase (SPS),^28−30^ the major breakthrough came from identifying the solanapyrone biosynthetic gene cluster (BGC) in *A. solani*.^31^ Using degenerate primers, real-time polymerase chain reaction (RT-PCR), and PCR-based genome walking, the solanapyrone (*sol*) BGC was identified as containing six genes, including a highly reducing polyketide synthase (HRPKS; *sol1*), an *O*-methyltransferase (OMT; *sol2*), an alcohol dehydrogenase (ADH; *sol3*), a transcription factor (TF; *sol4*), an oxidase (OXD; *sol5*), and a cytochrome P450 monooxygenase (P450; *sol6*). Homologous BGCs have also been identified in *A. rabiei* and *Peroneutypa* sp. M16, which are confirmed producers of solanapyrones A–C and solanapyrones S (**30**) and T (**31**), respectively (Figure 2).^32,33^

**Scheme 5:**
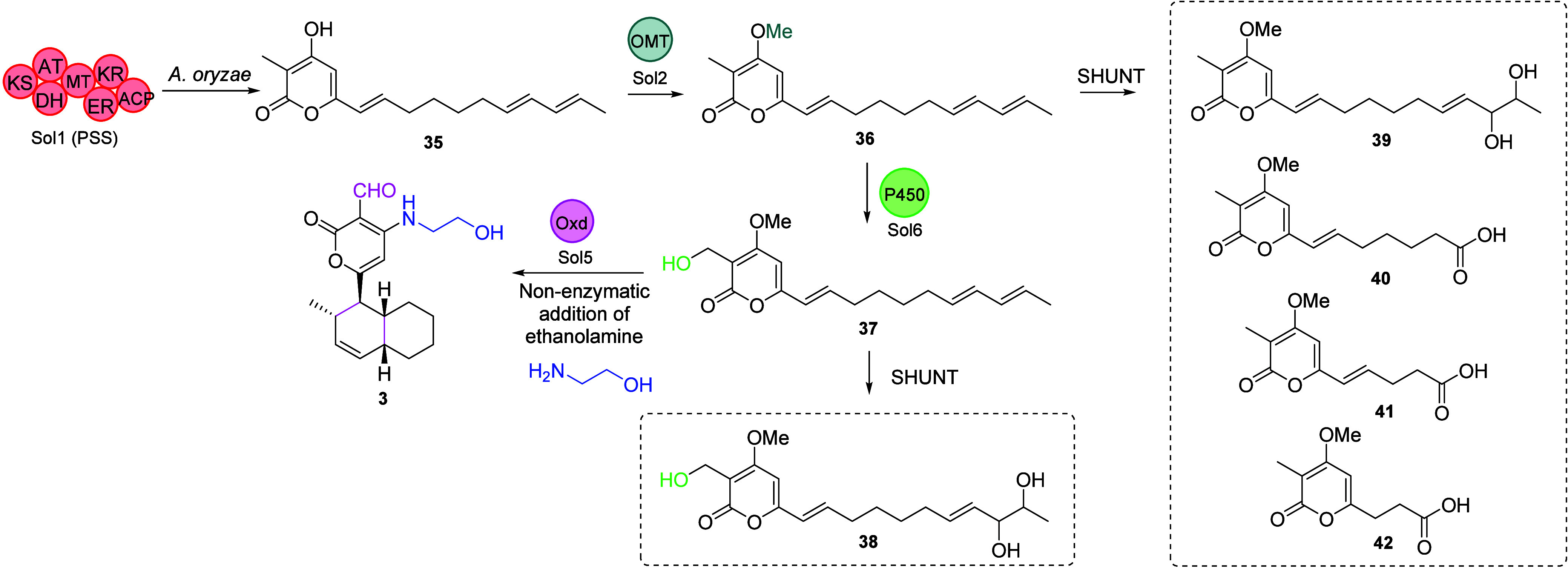
Biosynthetic Pathway of Solanapyrones Based on Gene Deletion and Heterologous Expression Studies^35^, Off-pathway intermediates arising from these investigations are shown in the dashed boxes.

Functional studies of *sol5* in both *A. solani* and *A. rabiei* via gene deletion resulted in the production of solanapyrones A–C (**1**–**3**) being abolished, and instead prosolanapyrone II-diol (**38**) accumulated (Scheme 5).^31^ Although deletion of *sol5* did not alter the pathogenicity of the fungi nor the growth rate or spore production, other genes in the *sol* BGC were overexpressed as a result.^32^ Gene disruption of *sol4* in *A. rabiei*, a putative Zn(II)2Cys6 transcription factor, also resulted in a loss of solanapyrone production; the overexpression led to increased expression levels of all genes in the BGC except *sol3*, indicating that *sol4* is a positive regulator of the sol BGC.^34^

Heterologous expression of *sol1* in *Aspergillus oryzae* led to the identification and purification of the octaketide desmethylprosolanapyrone I (**35**) (Scheme 5), therefore establishing HRPKS as prosolanapyrone synthase (PSS). The domains present within PSS are typical of a HRPKS, e.g., ketosynthase (KS), acyl transferase (AT), dehydrogenase (DH), ketoreductase (KR), enoyl reductase (ER), and acyl carrier protein (ACP). PSS lacks a domain obviously involved in the polyketide chain release. Instead, pyrone formation drives the release of the octaketide from the ACP domain. The biosynthetic genes *sol1/2/5/6* were heterologously expressed in *A. oryzae* in a stepwise fashion, enabling the role of each enzyme to be established (Scheme 5). Coexpression of *sol1* and *sol2* led to the production of prosolanapyrone I (**36**), confirming the role of the OMT as methylating the hydroxy group of **35**. When *sol1*, *sol2*, and *sol6* were coexpressed, prosolanapyrone II (**37**) was isolated, confirming the cytochrome P450 monooxygenase was responsible for the hydroxylation of the methyl group in **36**. Finally, when *sol1/2/5/6* were coexpressed solanapyrone C (**3**) was isolated. Because solanapyrone C (**3**) features an ethanolamine moiety, which was previously shown to be introduced nonenzymatically,^10^ the role of the OXD could be inferred as oxidizing the primary alcohol at C-14 to an aldehyde and catalyzing the intramolecular [4 + 2] cycloaddition reaction to generate solanapyrone A (**1**) from **37**. Solanapyrone synthase (SPS), encoded by *sol5*, is predicted to utilize a covalently bound flavin cofactor, which concurrently oxidizes a substrate while reducing O_2_ to H_2_O_2_.^31^ A series of shunt products and nonenzymatically generated products were also observed (**39**–**42**), highlighting the challenges of using heterologous expression to study biosynthetic pathways.^35^

## Characterizing Enzymatic [4 + 2] Cycloadditions Involved in the Biosynthesis of Solanapyrones

**Scheme 6:**
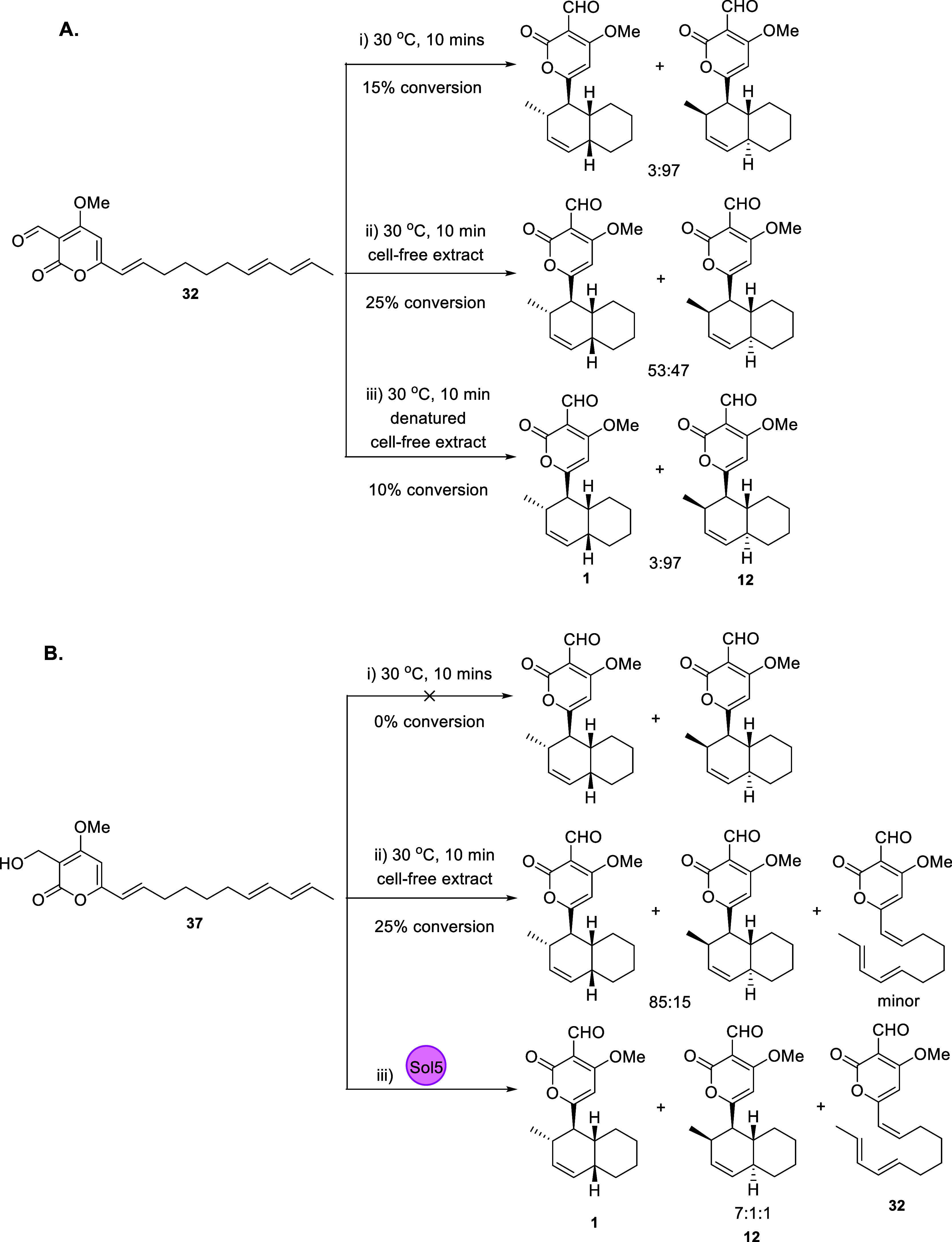
Overview of Enzymatic and Nonenzymatic Conversions of **32** and **37***In Vitro*(29), Observed major and minor products are indicated.

Initial investigations into the [4 + 2] cycloaddition reaction during solanapyrone biosynthesis utilized biosynthetic intermediates and cell-free extracts. The reactivity of prosolanapyrone III (**32**) was investigated in H_2_O, indicating that the *endo* product was the major product, although the substrate remained (mostly) unchanged (Scheme 6Ai).^29^ Prior to knowledge of the solanapyrone BGC, investigations into the putative [4 + 2]-cycloaddition enzyme used a partially purified cell-free extract from *A. solani*, which could convert 25% of prosolanapyrone III (**32**) into solA/D *exo*-selectively (53:47 *exo*/*endo* ratio) (Scheme 6Aii).^29^ When the denatured enzyme was used in the reaction, only 10% of **32** was converted with 3:97 selectivity (Scheme 6Aiii). When four times more enzyme was used, the reaction went four times faster. The apparent increase in reaction rate and control over the enantiomers produced indicated that a dedicated enzyme is required to convert **31** to **1**.^29^

Similarly, the enantiomeric selectivity of prosolanapyrone II (**37**) was also investigated. In H_2_O, **37** was not converted to **1** or **12** (Scheme 6Bi), whereas **1** and **12** were identified in a 85:15 ratio using cell-free extract, with a small amount of prosolanapyrone III (**32**) being generated (Scheme 6Bii). Prosolanapyrone II **37** was therefore proposed to be oxidized to prosolanapyrone III (**32**), which is the preferred substrate for cycloaddition.^29^ Further experiments determined that molecular oxygen was essential for the enzymatic activity and generated H_2_O_2_. When O_2_ was replaced with argon, SPS could not convert prosolanapyrone II (**37**), further supporting the finding that oxidation of **37** to **32** occurs prior to cyclization.^29^ The stoichiometry of oxidation of alcohol **37** to aldehyde **32** indicated than an NAD(P)H independent oxidase was required.^10^

**Scheme 7:**
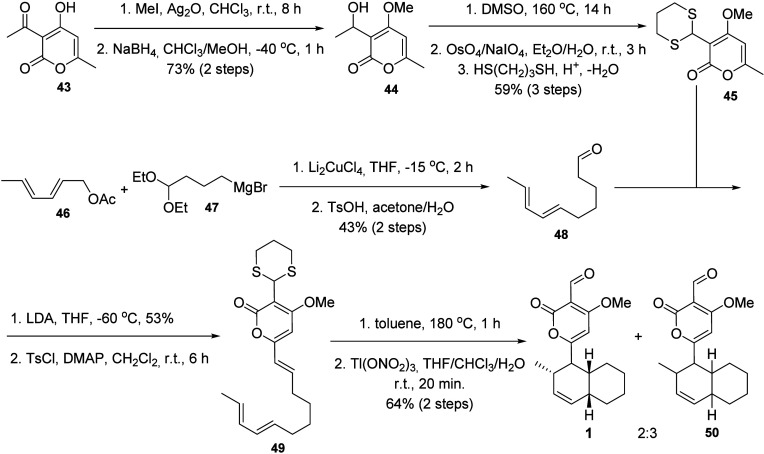
First Total Synthesis of Solanapyrone A (**1**)^24^

**Scheme 8:**
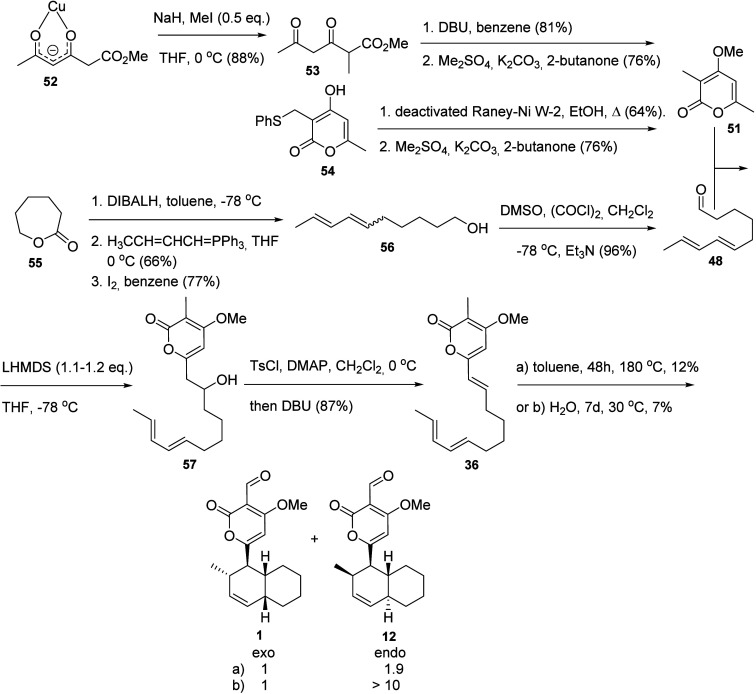
Initial Steps in the Detailed Investigation of the Synthesis of Solanapyrones: Synthesis of Solanapyrone A (**1**) and Solanapyrone D (**12**)^25^

**Scheme 9:**
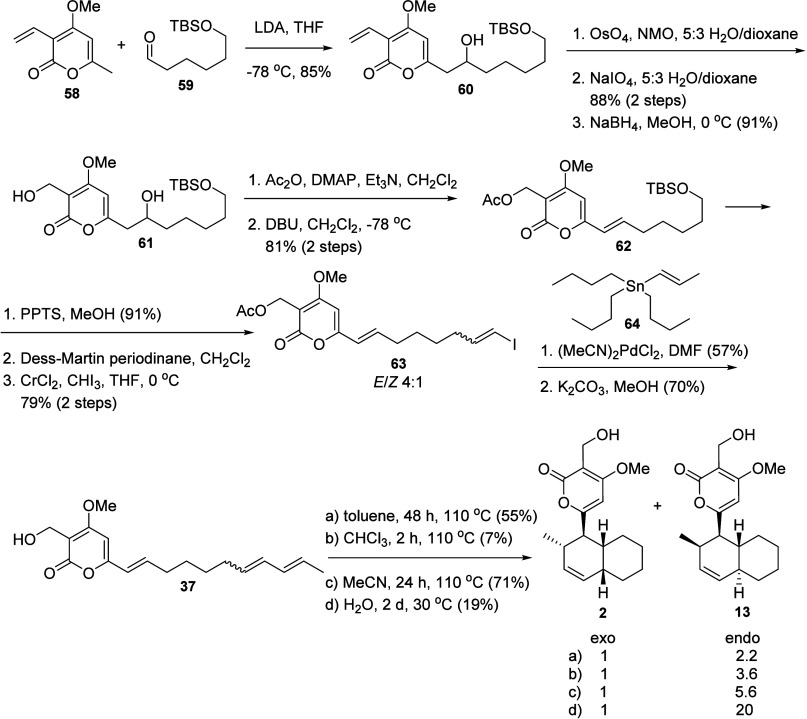
Next Steps in the Detailed Investigation of the Synthesis of Solanapyrones: Synthesis of Solanapyrone B (**2**)^25^

**Scheme 10:**
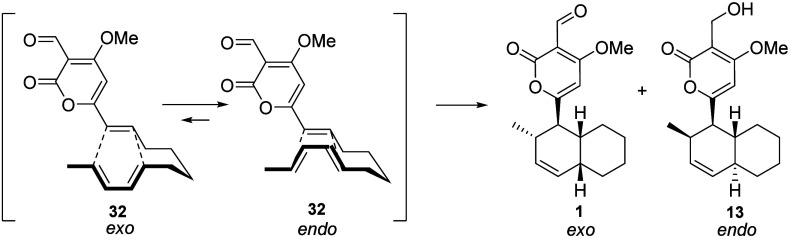
Transition States (*exo*-**32** and *endo*-**32**) for the Diels–Alder Reaction Towards the Formation of Solanapyrones A (**1**) and E1 (**13**)^25^

In the course of the synthesis of solanapyrones A (**1**) and B (**2**) (Schemes 7–10, see below), the enzymatic conversion of prosolanapyrones into solanapyrones was performed using a crude Diels–Alderase enzyme,^25^ confirming the earlier assumptions.^11^ A later study provided the detailed procedure on how the enzyme was purified.^28^ The enzymatic reaction with **37** as the substrate yielded solanapyrone A (**1**) with 99% enantiomeric excess and 6:1 *exo/endo* selectivity, reversing the selectivity of the nonenzymatic procedure. However, at higher concentrations of the substrate the reaction lost both enantio- and stereoselectivity, likely caused by conversion of the hydroxymethylene group in **37** into its corresponding aldehyde, a substrate that favors the nonenzymatic formation of the unnatural *endo* product. Attempts to overcome the loss of selectivity at high substrate concentrations involved freezing the freshly prepared crude enzyme in 30% glycerol. The recovery of products after the enzymatic Diels–Alder reaction also proved challenging, being solved by extracting the reaction medium with HP-20 and desorption of the reaction products with EtOAc. By using such optimized conditions, a 5:1 mixture of the *E*/*Z* stereoisomers of **37** was converted into solanapyrones A (**1**) and D (**12**) in 61% yield and >98% and 67% ee, respectively. Under such conditions, about 10% of the corresponding aldehyde mixture formed from **37** was recovered from the enzymatic reaction. From the outcome of the reaction, the authors propose that the enzyme first converts the *E*/*Z* isomers of **37** to the corresponding aldehydes before the Diels–Alder reaction, claiming that the formation of a racemic mixture of solanapyrone D (**12**) is nonenzymatic. The main outcome of this very elegant work was the discovery of the first enzyme-catalyzed Diels–Alder reaction.^25^

Recombinant expression of Sol5 (SPS) in *E. coli* did not lead to active enzyme despite high-levels of protein being produced, similar to attempts to isolate SPS from *A. oryzae* expression strains.^31^ The fungal host *Pichia pastoris* was utilized instead; *sol5* cDNA lacking the secretion signal was expressed without a His-tag.^31^ The *P. pastoris* culture medium was verified to convert **37** to **1**, indicating the functional enzyme, and SPS was purified from 1 L of the induced culture medium. Prosolanapyrone II (**37**) was incubated with purified SPS to yield compounds **1**, **12**, and **32** in a 7:1:1 ratio (Scheme 6Biii), confirming its role as a bifunctional oxidase and in catalyzing the [4 + 2] cycloaddition reaction.^31^

While Diels–Alderases required for the biosynthesis of several other decalin-containing natural products have been extensively investigated biochemically and structurally in recent years,^36−39^ there are still some doubts over SPS.^40,41^ Although the *in vivo* and *in vitro* biochemical investigations convincingly show that SPS facilitates the [4 + 2] cycloaddition, it is unknown whether formation of the decalin core is truly a pericyclic mechanism as opposed to a stepwise and nonconcerted mechanism. So far, no crystal structure of SPS has been reported and thus the amino acid residues that facilitate the [4 + 2] cycloaddition reaction are unknown.

## Total Synthesis of Solanapyrones

As soon as the first solanapyrones were isolated and identified, different approaches were developed for the total synthesis of these metabolites. The key steps were the formation of the dehydrodecalin system and that of the pyrone moiety.

The first total synthesis of solanapyrone A (**1**) (Scheme 7)^24^ started with the methylation and subsequent reduction of substrate **43** to give the alcohol **44**. Dehydration of **44** in DMSO followed by double bond oxidation provided an olefin, which was oxidized to an aldehyde and protected with propane-1,3-dithiol to give **45** in 43% overall yield over five steps. In parallel, the diene aldehyde **48** was prepared from the condensation between (2*E*,4*E*)-hexa-2,4-dien-1-yl acetate (**46**) and (4,4-diethoxybutyl)magnesium bromide (**47**) in the presence of Li_2_CuCl_4_ to give the extended diethylketal, which was hydrolyzed to the corresponding aldehyde (**48**) in 43% overall yield. Aldol condensation of **48** with **45** followed by the corresponding intermediate alcohol dehydration gave triene **49**, the configuration of which was confirmed by NMR analysis. The triene **49** was subjected to an intramolecular Diels–Alder reaction to provide a 1:2 mixture of stereoisomers of the corresponding protected aldehydes, where the minor isomer corresponded to the correct *cis*-stereoisomer at the dehydrodecalin moiety. As this mixture proved to be inseparable, it was subjected to dithiane deprotection to give **1** as well as a diastereoisomer **50**, for which the relative configuration at the dehydrodecalin moiety junction was not established.^24^

The first synthesis of **1** was subject to a very detailed optimization investigation (Schemes 8–10), which included an enzymatically catalyzed Diels–Alder cyclization step and the first description of an enzyme-catalyzed Diels–Alder reaction.^25^ An initial approach toward the pyrone moiety **51** started with the methylation of the copper complex **52** to give **53** in 88% yield, followed by cyclization to **51** in overall 61% yield. An alternative procedure to **51** was developed from thioether **54** (previously prepared) in 43% yield. The preparation of aldehyde **48** was also improved to a 63% yield procedure via the reduction of caprolactone (**55**) to its corresponding aldehyde, followed by a Wittig reaction with crotyl phosphonium to give the alcohol **56** in 66% yield for two steps. Improving the stereoselectivity for the formation of the conjugated diene was possible through treatment of the product of the Wittig reaction with I_2_ (from 1:1 to 5:1 *E*,*E*- and *E*,*Z*-isomeric dienols, respectively). Swern oxidation provided dienal **48**. Condensation of **51** with **48** followed by Ec2b elimination gave **36** via **57** in 87% yield (2 steps). Intramolecular Diels–Alder cyclization of **36** using two different conditions was attempted but resulted in poor yields and poor stereoselectivity to obtain solanapyrone A (**1**) and solanapyrone D (**12**) (Scheme 8).

Next, a second approach, toward solanapyrone B (**2**) (Scheme 9), began with an aldol condensation of **58** and the aldehyde **59** to give the alcohol **60**. The terminal double bond was then oxidized with OsO_4_/NMO and cleaved with NaIO_4_, and the resulting aldehyde was reduced to **61** with NaBH_4_. Acetylation of both alcohol functionalities of **61** followed by an E2cB elimination of the alkyl chain acetate provided **62** in 81% yield for 2 steps. The terminal long-chain alcohol was then deprotected with PPTS, oxidized to an aldehyde with Dess-Martin periodinane, and subjected to the Takai reaction with CHI_3_ to provide **63** as a mixture of *E*/*Z* isomers, with a predomination of the *E* stereoisomer. A Stille reaction between mixture **63** with (1*E*)-propenylSnBu_3_ (**64**) and (MeCN)_2_PdCl_2_ in DMF yielded **37** after alkaline hydrolysis as a mixture of *E*/*Z* isomers near the end of the long chain. Four different conditions [a–d] were attempted for the nonenzymatic Diels–Alder reaction of **37**, yielding the undesired *endo* diastereomer solanapyrone E1 (**13**) of solanapyrone B as the major product under each condition. The results obtained were explained in terms of the substituent at the pyrone ring, either methyl, hydroxymethylene, or aldehyde, in the aqueous medium considering the two transition states (**32***exo* and **32***endo*) for the Diels–Alder reaction and the solvent effect (Scheme 10). The authors indicated that an electron-withdrawing group (CHO) would favor the formation of the *endo* adduct by either hydrophobic or both hydrophobic and hydrogen effects in H_2_O, which was explained by the frontier orbital theory for [4 + 2] cycloadditions. Thus, the authors selected the mixture of stereoisomers **37** for an enzymatic conversion to natural solanapyrone B (**2**), aiming to minimize the nonenzymatic reaction that may reduce the stereoselective formation of solanapyrone B.^25^

**Scheme 11:**
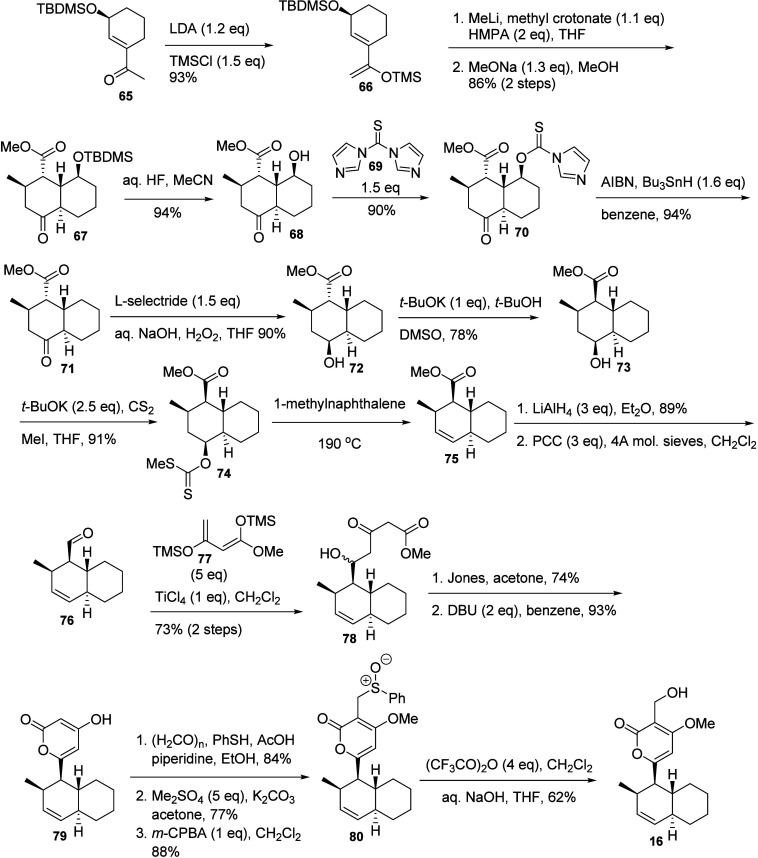
Total Synthesis of Solanapyrone G (**16**)^42,43^

Some confusion between names of solanapyrones E2 (**14**) and G (**16**) was found in the title of papers reporting the total synthesis of solanapyrone E2 (**14**) because, in fact, the correct name of the synthesized compound is solanapyrone G (**16**).^42,43^ Synthesis of solanapyrone G (**16**) was achieved by a completely distinct strategy employing a domino Michael approach (Scheme 11).^42^ The starting substrate (**65**) was previously obtained by lipase-catalyzed enantioselective acylation. Formation of the protected enol (**66**) was followed by the domino Michael reaction with methyl crotonate and treatment with a base toward the isomerization to the thermodynamically stable *trans*-decalone **67**, characterized by NMR analysis. The TBDMS-protected alcohol group in **67** was removed from the bicyclic framework by deprotection, reaction with thiocarbonyldiimidazole (**69**), and reductive treatment with *n*-tributyltin hydride to give **71** via **68** and **70**. The ketoester **71** was then reduced to **72**, and the ester group subjected to epimerization to give **73** before activation of the alcohol group to form corresponding xanthate **74**, which was subjected to an elimination reaction to give **75**. The product **75** was directly reduced with LiAlH_4_, and the resulting product was oxidized with PCC to the corresponding aldehyde **76**. Aldehyde **76** was reacted with bistrimethylsilyl enol ether of methyl acetoacetate (**77**) to give the δ-hydroxy-β-ketoester **78**, which was directly cyclized into the pyrone **79**. Installation of the hydroxylmethylene group was performed by reaction with paraformaldehyde and thiophenol. Then the enol group was methylated, the phenyl sulfide was oxidized with *m*-CPBA, and the resulting sulfoxide **80** was reacted with triflate anhydride, followed by treatment with base, to afford solanapyrone G (**16**) in overall 6% yield. A full account on this strategy was later published,^43^ which included the synthesis of solanapyrone D (**12**) from the sulfoxide **80** via reaction with trimethylsilyltriflate and trimethylsilyldiethylamine, followed by treatment with tetrabutylammonium fluoride in 69% yield.

**Scheme 12:**
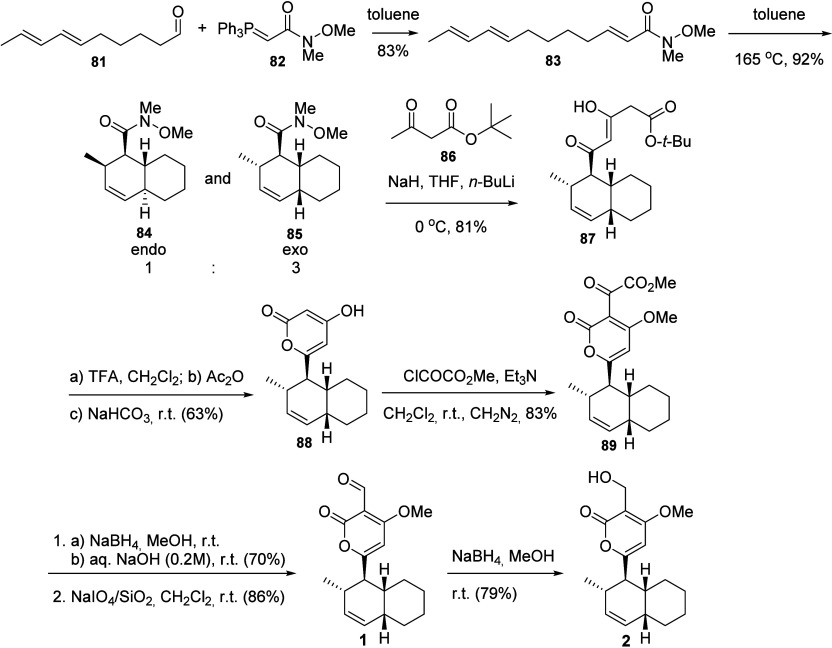
Total Synthesis of Solanapyrones A (**1**) and B (**2**) via Diels–Alder Reaction of the Weinreb Amide **83**(44)

An alternative approach toward solanapyrones involved an investigation of the intramolecular Diels–Alder reaction with different electron-withdrawing groups attached to the dienophile toward the formation of the dehydrodecalin bicyclic moiety (Scheme 12).^44^ This synthesis started with a Wittig reaction between the unsaturated aldehyde **81** (previously prepared^45^) with the phosphorane of the Weinreb amide **82** to provide the amide **83**. The substrate **83** was selected for the Wittig reaction among several carbonyl-attached groups because the Weinreb-substituted dienophile of **83** performed better for the [4 + 2] cycloaddition reaction in terms of both chemical yield and stereoselectivity, favoring the formation of the desired *exo*-adduct (**85**). Both cycloaddition products **84** and **85** were separated by chromatography. Condensation of **85** with the dianion of *t*-butyl acetoacetate (**86**) provided the ester **87**, which was converted into the dehydrodecalin-substituted pyrone **88**. Acylation of **88** with methyl oxalyl chloride in the presence of Et_3_N provided α-ketoester **89**. Substrate **89** was converted into solanapyrone A (**1**) by conversion to its α-hydroxy acid and then oxidized to **1**. Solanapyrone B (**2**) was also prepared by NaBH_4_ reduction of solanapyrone A (Scheme 12).^44^

**Scheme 13:**
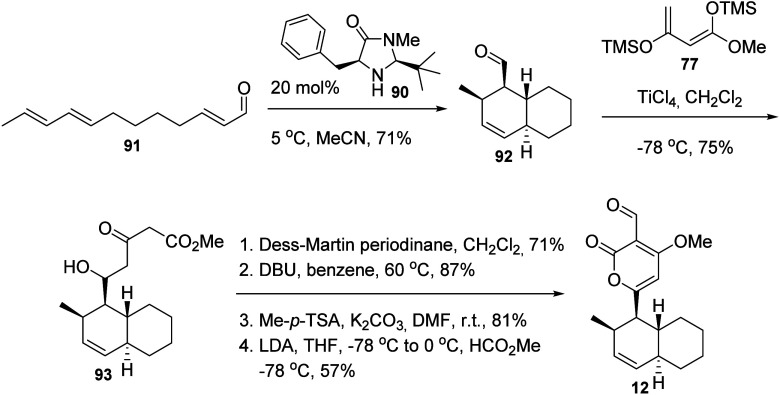
Total Synthesis of Solanapyrone D (**12**) Using the Second-Generation Imidazolinone Catalyst **90**(46)

The last synthesis developed for solanapyrones was achieved by the group of Nobel Prize in Chemistry awardee David W. C. MacMillan (Scheme 13).^46^ Using a “second generation” of the imidazolinone catalyst **90**, the intramolecular Diels–Alder reaction of the conjugated aldehyde **91** was achieved in 71% yield, 90% ee and >20:1 diastereoselectivity favoring the *endo* product **92**. The cycloaddition product **92** was condensed with the previously employed bistrimethylsilyl enol ether of methyl acetoacetate (**77**; see above) to give the aldol product **93**. After cyclization of **93** into its corresponding substituted pyrone, the product was methylated and acylated to give solanapyrone D (**12**) in only six steps and in 25% overall yield.

## Biological Activities of Solanapyrones A–C

The biological activities of the solanapyrones have been extensively investigated. Because solanapyrones A–C (**1**–**3**) were originally isolated from cultures of *Alternaria solani*, the fungus responsible for the blight of tomato and potato, the compounds were thought to be the phytotoxins associated with the disease. In the original isolation report of solanapyrones A–C,^6^ it is mentioned that solanapyrone A (**1**) caused lesions on the leaves of potato at the concentration of 100 μg/100 μL (i.e., 1 mg/mL). However, this concentration is considered rather high for compounds expressing phytotoxic activity.^47^ Solanapyrones were subsequently reported from cultures of *Ascochyta rabiei*, a fungus strain that causes blight on chickpea and has been known for almost 100 years.^48^ When tested on different cultivars of chickpea, solanapyrones A (**1**) and C (**3**) displayed variable phytotoxic activity, between 3.7 and 17.1 μM for **1** and between 14.1 and 74.2 μM for **3**.^8^ When applied as a 1:1:0.2 mixture of solanapyrones A–C (**1**–**3**) on chickpea leaflets, the plant showed symptoms detected by fluorescence microscopy at 200 μM. Solanapyrone concentrations between 100 and 200 μM induced chlorophyll bleaching on the spot of the sample application and, subsequently, white spots were observed.^13^ At higher concentrations (1–2 mM), the white spots turned to a brown color and suffered tissue disruption. When treated with the same mixture of solanapyrones, heterotrophic cell suspension cultures derived from chickpeas were sensitive to a 100 μM concentration of the mixture, with cells showing plasmolysis and protoplasts aggregation.^13^ Cell death was observed with application of a 200 μM solution of solanapyrones mixture. However, solanapyrones were not detected in plant tissues infected with *A. rabiei*.^13^ In another investigation, nine strains of *A. rabiei* were investigated for the production of solanapyrones.^48^ The production of solanapyrones A–C (**1**–**3**) varied considerably among strains, some of which did not produce these compounds under the same conditions. One of the strains produced cytochalasin D, which also affected chickpea cuttings with similar symptoms as those caused by **1**–**3**. The response of chickpea cultivars to different *A. rabiei* strains was not consistent, raising a question regarding the phytotoxicity of solanapyrones **1**–**3**.^48^

In chickpea seedling root growth inhibition assays,^49^ solanapyrone A (**1**) was the most active, at 250 μM, followed by solanapyrone B (**2**) at 450 μM and solanapyrone C (**3**) at 600 μM. While the activity of solanapyrones A and C produced by *A. solani* on tomatoes and potatoes were observed as synergistic only, the activity of the same compounds produced by *A. rabiei* on chickpeas was additive.^49^ The results above showed the individual activity of solanapyrones A–C (**1**–**3**) on chickpea seedling roots. However, different chickpea cultivars showed different responses in the same assay, indicating some level of inconsistency in the results obtained in this assay.^49^

Chickpea shoots incubated with solanapyrone A (136 μg/mL) became shriveled, brown, and corky, while leaflets presented flame-shaped chlorotic zones.^50^ Prolonged incubation with solanapyrone A caused bleaching of the chickpea stems. However, the authors observed that solanapyrones A and B degraded in H_2_O, an unusual result considering that the fungus producing solanapyrones was grown in aqueous media. Chickpea cultivars were killed with a solanapyrone concentration of 10 μg/mL. Chickpea cultivars were much more sensitive to the growth in the presence of solanapyrone A than in the presence of solanapyrone B; solanapyrone A was 2.5–12.5 times more toxic to chickpea cultivars than solanapyrone B depending on the cultivar used in the assay. The authors mention that solanapyrones caused epinasty, chlorosis, necrosis, and breakage on chickpea stems but did not provide data to support the claims. It is worth mentioning that considerable variations in the assays were observed from day to day, indicating that further investigations on the phytotoxicity of solanapyrones are necessary.^50^

Solanapyrone A also inhibited rat DNA polymerase β and human DNA polymerase λ, but did not inhibit replicative DNA polymerases.^51^ The 50% inhibitory concentration for DNA polymerase β was 30 μM, and complete inhibition occurred at 80 μM, but solanapyrone A inhibited other DNA polymerases at higher concentrations and was not active as inhibitor of prokaryotic DNA polymerases.^51^ Solanapyrone A promoted necrosis of tomato leaves after 5 days of the treatment at concentrations between 0.1 and 0.25 μM.^52^ Solanapyrone A also induced phosphorylation of RiCDPK2, an enzyme that is an isoform of calcium-dependent protein kinases occurring in potatoes. The effect was observed at 25 μM **1**, promoting a 43% increase in phosphorylation, and was maintained for 40 min.^52^

However, the phytotoxicity of solanapyrones was questioned in subsequent investigations,^32,53^ which demonstrated that chickpea blight had no direct correlation with the presence of solanapyrones produced by *A. rabiei*.^32^ Therefore, the actual biological activity of the solanapyrones remains elusive.

## Perspectives on the Chemistry, Biochemistry, and Biology of Solanapyrones

Solanapyrones belong to a small, but growing, group of secondary metabolites presenting a 1,2-disubstituted Δ^3^-dehydrodecalin core arising from an enzymatically catalyzed Diels–Alder cycloaddition or from a formal [4 + 2] cycloaddition that resembles a Diels–Alder reaction. Also belonging to this group are the HMG-CoA inhibitors lovastatin, simvastatin, compactin, and pravastatin;^54^ several metabolites presenting a tetramic acid moiety,^55^ such as the HIV-integrase inhibitors and phytotoxins equisetin and trichosetin;^56^ paecilosetin and derivatives^57^ as antimicrobial compounds; the antibiotic cissetin-A;^58^ CJ-21,058;^59^ ascosalipyrrolidinone B, an anti-*Trypanosoma cruzi* agent;^60^ LL-F49&233a, which displayed antibiotic activity against various human bacterial pathogens;^61^ beauversetin;^62^ the mild antibiotics zopfiellamides A and B;^63^ lydicamycin, along with four additional related derivatives that are antibiotics and the most complex members of this family of metabolites;^64^ coprophilin, an antiprotozoan agent produced by an unidentified fungus strain;^65^ and deoxynortrichoharzin.^66^

The large majority of these metabolites are produced by fungi. Bacterial metabolites bearing the same cycloaddition-derived 1,2-disubstituted Δ^3^-dehydrodecalin moiety include integramycin produced by *Actinoplanes* sp. ATCC202188^67^ and kibdelomycin and several derivatives, which are very potent antibiotic compounds.^68^ Related metabolites have been discovered from both fungal and bacterial cultures.^69^ Along with the bioactivity reported for the majority of such compounds, and the growing interest in the total synthesis of these metabolites,^70^ their unique biosynthesis, via a Diels–Alderase or a functionally similar enzyme leading to the dehydrodecalin moiety, is a subject of increasing investigation because of its intrinsic interest and also because of its potential application in organic synthesis biocatalysis.^55,71−75^

While the actual bioactivity of solanapyrones remains to be discovered, these are the only metabolites of this group bearing a fully oxidized lactone group containing a pyrone. Whether such a structural feature is related to the moderate or nonexistent bioactivity of solanapyrones is not yet known. It is possible that these compounds represent a metabolic diversification of these metabolites resulting from an evolutionary branch arising from a mutation that fully oxidizes the polyketide chain connected to C-1, leading to metabolites that are less biologically active than less oxidized derivatives.^54−66^ However, this is a hypothesis that remains to be investigated in light of the biosynthesis of these metabolites. Certainly, further research on the biosynthesis of solanapyrones and on the above related metabolites may shed light on the evolution of Diels–Alderases in fungi and their potential application as biocatalysts.

## Dedication

Dedicated to Dr. Sheo B. Singh, retired from Merck, now with Drew University and Stevens Institute of Technology, for his pioneering work on bioactive natural products.
